# Supplementary material for: Curated and harmonised transcriptomics datasets of interstitial lung diseases
Source: Data Brief. 2025 Oct 14;63:112139. doi: 10.1016/j.dib.2025.112139 (PMC12581653; doi:10.1016/j.dib.2025.112139)
Supplement: Supplementary file 1 [file mmc1.zip › Supplementary_material/RNA-seq/fastQC reports/GSE184316/SRR15926752_fastqc.html]

SRR15926752.fastq.gz FastQC Report 

FastQC Report

Mon 5 Feb 2024  
SRR15926752.fastq.gz

## Summary

- Basic Statistics
- Per base sequence quality
- Per sequence quality scores
- Per base sequence content
- Per sequence GC content
- Per base N content
- Sequence Length Distribution
- Sequence Duplication Levels
- Overrepresented sequences
- Adapter Content

## Basic Statistics

| Measure | Value |
| --- | --- |
| Filename | SRR15926752.fastq.gz |
| File type | Conventional base calls |
| Encoding | Sanger / Illumina 1.9 |
| Total Sequences | 9588006 |
| Sequences flagged as poor quality | 0 |
| Sequence length | 25-371 |
| %GC | 48 |

## Per base sequence quality

## Per sequence quality scores

## Per base sequence content

## Per sequence GC content

## Per base N content

## Sequence Length Distribution

## Sequence Duplication Levels

## Overrepresented sequences

| Sequence | Count | Percentage | Possible Source |
| --- | --- | --- | --- |
| GGAAGACAGTGTGGCGATTCCTCAGGGATCTAGAACTAGAAATACCATTG | 153362 | 1.5995192326746561 | No Hit |
| ATTGATTCTTCCTACCCATGAGCATGGAATGTTCTTCCATTTGTTTGTAT | 120243 | 1.2540980887996942 | No Hit |
| GAAGGACCTCTTCAAGGAGAACTACAAACCACTGCTCAAGGAAATAAAAG | 110559 | 1.1530969004399871 | No Hit |
| ATAGCAGCATGATTTATAGTCCTTTGGGTATATACCCAGTAATGGGATGG | 96532 | 1.0067995368380036 | No Hit |
| GAAGGACCTCTTCAAGGAGAACTACAAACCACTGCTCAATGAAATAAAAG | 77341 | 0.8066432165353256 | No Hit |
| ATAGCAGCATGATTTATAATCCTTTGGGTATATACCCAGTAATGGGATGG | 57174 | 0.596307511697427 | No Hit |
| GCATGTGTCTTTATAGCAGCATGATTTATAGTCCTTTGGGTATATACCCA | 32021 | 0.3339693362728392 | No Hit |
| GGCCATTTTCATGATATTGATTCTTCCTACCCATGAGCATGGAATGTTCT | 30523 | 0.3183456497628391 | No Hit |
| ATTGATTCTTCCTACCCATGAGCATGGAATGTTCTTCCATTTGTTTGTGT | 29294 | 0.3055275518183864 | No Hit |
| GGAAGACAGTGTGGCGATTCCTCAAGGATCTAGAACTAGAAATACCATTG | 28355 | 0.2957340660821447 | No Hit |
| CAGTGGGGCCACGAGCTGAGTGCGTCCTGTCACTCCACTCCCATGTCCCT | 23154 | 0.2414892105824715 | No Hit |
| GCATGTGTCTTTATAGCAGCATGATTTATAATCCTTTGGGTATATACCCA | 22191 | 0.23144541211175712 | No Hit |
| GGAAGACAGTGTGGTGATTCCTCAGGGATCTAGAACTAGAAATACCATTG | 20629 | 0.21515422497649667 | No Hit |
| AGGCTACACACTGAGGACTCTGTTCCTCCCCTTTCCGCCTAGGGGAAAGT | 20045 | 0.20906328177099598 | No Hit |
| GGAAGACAGTGTGGCGATTCCTCAGGGATCTAGAACTAGAAATACCATTT | 19832 | 0.20684175625255136 | No Hit |
| CAATCCACTTGATCCCAACTCATCTCTCATTTATTTCGGCTTCTTTTATT | 19358 | 0.20189807974671686 | No Hit |
| ATAGCAGCATGATTTATAGTCATTTGGGTATATACCCAGTAATGGGATGG | 19113 | 0.1993428039156421 | No Hit |
| GGAAGACAGTGTGGCGATTCCTCAGGGATCTAGAACTGGAAATACCATTG | 18047 | 0.18822474662614938 | No Hit |
| TGATATGGTTCCTGGCAAGCCCATGTGTGTTGAGAGCTTCTCAGACTATC | 17531 | 0.1828430228349878 | No Hit |
| CACCTCCCCGAAGCTCAGGGAGAGCCCTGTTAGGGCCGCCTCTGGCCCTA | 17210 | 0.17949509001141636 | No Hit |
| ATTGATTCTTCCTATCCATGAGCATGGAATGTTCTTCCATTTGTTTGTGT | 17169 | 0.17906747242335894 | No Hit |
| TTCAGCCCTGATCAAGACATGAGGGAGGCAGGGGCTCAGCTGAAGAAGCT | 16869 | 0.17593856324245102 | No Hit |
| CTTCATGATGTAGCAGCAGGTGCCAGGGGCTGGCTTGTAGGCGATCAGCA | 16519 | 0.1722881691980585 | No Hit |
| GGAAGACAGTGTGGCAATTCCTCAGGGATCTAGAACTAGAAATACCATTG | 16313 | 0.17013965156050173 | No Hit |
| TACCACTGCCACCTTCTCCATCGGCTCCACTGGCCTCGTGGTGTATGACT | 15455 | 0.16119097130310514 | No Hit |
| GGGAAGCGGGGAATGTCTACGTGCGTATGCACGTGGCACTCTCTGCCCGA | 15215 | 0.15868784395837884 | No Hit |
| TGATGACACCCACCGCAACTGTCTGTCTCATATCACGAACAGCAAAGCGA | 15183 | 0.15835409364574868 | No Hit |
| GGAAGACAGTGTGGTGATTCCTCAAGGATCTAGAACTAGAAATACCATTG | 15033 | 0.1567896390552947 | No Hit |
| GAAGGACCTCTTCAAGGAGAACTACAAACCACTGCTCAACGAAATAAAAG | 14433 | 0.15053182069347892 | No Hit |
| TTACAAAAGCAGAATAAAAGCGAAAAGAAATGAAAATGTTACACTACATT | 14396 | 0.15014592189450027 | No Hit |
| GGAAGACAGTGTGGCAATTCCTCAAGGATCTAGAACTAGAAATACCATTG | 13079 | 0.1364100105903146 | No Hit |
| ATTGATTCTTCCTACCCATGAGCATGGAATGTTCTTCCATTTCTTTGTAT | 11925 | 0.1243741399410889 | No Hit |

## Adapter Content

Produced by FastQC (version 0.11.7)
